# A Review of the Metabolism and Relevance to Form and Formulation of Ketamine

**DOI:** 10.1192/j.eurpsy.2023.1783

**Published:** 2023-07-19

**Authors:** P. Chue, A. Andreiev, J. Chue, J. Chue, M. Tate

**Affiliations:** 1 University of Alberta, Edmonton; 2 University of Ottawa, Ottawa; 3 Oakville Trafalgar Memorial Hospital, Toronto; 4 Amygdala Associates, Edmonton, Canada

## Abstract

**Introduction:**

Ketamine is a phenylcyclohexylamine derivative comprising a racemic mixture of S- and R-ketamine that possesses anesthetic, analgesic, anti-inflammatory, and antidepressant activity. Oral (including extended release [PO]), intravenous (IV) sublingual (SL), transmucosal (TM), intranasal (IN), intramuscular (IM), rectal (PR), and subcutaneous (SC) formulations have been developed since its commercialization in 1970.

**Objectives:**

To review and understand the impact of different forms and formulations on the pharmacokinetics of ketamine.

**Methods:**

The extant literature on ketamine metabolism and formulations was reviewed and discussed.

**Results:**

IV (racemic) ketamine (KET) has been shown to improve depressed mood within 4 hours with maximal effect at 24 hours. KET is a chiral molecule with two optimal isomers, R- and S-KET. KET is stereoselectively metabolized by CYP2B6 and CYP3A4 initially via nitrogen demethylation to active metabolite, norketamine (NK); there is no interconversion between R- and S-KET. NK is further metabolized to hydroxynorketamine (HNK) by CYP3A4 and CYP3A5; and dehydronorketamine (DHNK) by CYP2B6. Additional metabolic pathways exist including a direct enantioselective hydroxylation of KET to 6-hydroxyketamine (HK). Bioavailablity is greatest (100%) with the IV racemic KET formulation, but as low as 8% for oral S-KET due to extensive first-pass metabolism; the KET: NK ratio is a measure of first pass metabolism. NK plasma levels are higher with oral S-KET than KET as a result of local intestinal metabolism effects. Additionally, greater plasma concentrations are noted with IV bolus doses of S-KET vs. racemic KET or R-KET. S-KET possesses a longer elimination half-life than racemic KET due to inhibition by R-KET. KET is primarily renally eliminated and twice as fast in children vs. adults.

**Conclusions:**

Complex interactions are reported between ketamine form (racemic/enantiomer), formulation, dose, and route of administration that impact on clinical variables and thus, outcome.

**Disclosure of Interest:**

None Declared

